# Flexophotovoltaic Effect in Potassium Sodium Niobate/Poly(Vinylidene Fluoride‐Trifluoroethylene) Nanocomposite

**DOI:** 10.1002/advs.202004554

**Published:** 2021-02-08

**Authors:** Chenchen Wang, Yang Zhang, Bowen Zhang, Bo Wang, Jinxi Zhang, Long‐Qing Chen, Qiming Zhang, Zhong Lin Wang, Kailiang Ren

**Affiliations:** ^1^ Beijing Key Laboratory of Micro‐nano Energy and Sensor; CAS Center for Excellence in Nanoscience Beijing Institute of Nanoenergy and Nanosystems, Chinese Academy of Sciences Beijing 101400 P. R. China; ^2^ Institute of Semiconductors Chinese Academy of Sciences Beijing 100083 P.R. China; ^3^ Department of Materials Science and Engineering The Pennsylvania State University University Park PA 16802 USA; ^4^ Department of Electrical Engineering and Materials Research Institute Pennsylvania State University University Park PA 16802 USA; ^5^ School of Material Science and Engineering Georgia Institute of Technology Atlanta GA 30332 USA; ^6^ School of Physical Science and Technology Guangxi University Nanning Guangxi 530004 P.R. China

**Keywords:** flexophotovoltaic, nanocomposites, photovoltaic, piezoelectricity

## Abstract

Flexoelectricity is an electromechanical coupling effect in which electric polarization is generated by a strain gradient. In this investigation, a potassium sodium niobite/poly(vinylidene fluoride‐trifluoroethylene) (KNN/PVDF‐TrFE)‐based nanocomposite is fabricated, and the flexoelectric effect is used to enhance the photovoltaic current (*I*_pv_) in the nanocomposite. It is found that both a pyroelectric current and photovoltaic current can be generated simultaneously in a light illumination process. However, the photovoltaic current (*I*_pv_) in this process contributes ≈85% of the total current. When assessing the effect of flexoelectricity with a curvature of 1/20, the *I*_pv_ of the curved KNN/PVDF‐TrFE (20%) (K/P‐20) composite increased by ≈13.9% compared to that of the flat K/P‐20 nanocomposite. Similarly, at a curvature of 1/20, the *I*_pv_ of the K/P‐20 nanocomposite is 71.6% higher than that of the PVDF‐TrFE film. However, the photovoltaic effect induced by flexoelectricity is much higher than the increased polarization from flexoelectricity, so this effect is called as the flexophotovoltaic effect. Furthermore, the calculated energy conversion efficiency of the K/P‐20 film is 0.017%, which is comparable to the previous research result. This investigation shows great promise for PVDF‐based nanocomposites in ferroelectric memory device applications.

## Graphical abstract

Here, a potassium sodium niobite/PVDF‐TrFE nanocomposite is utilized to enhance the photovoltaic current (*I_pv_*) of the nanocomposite. It is found that photovoltaic current in this process contributes ≈85% of the total current. Compared to the flat nanocomposite, flexoelectricity (curvature: 1/20) can increase the *I_pv_* of the nanocomposite by 13.9%. This is the first investigation on the flexophotovoltaic effect of PVDF nanocomposites.

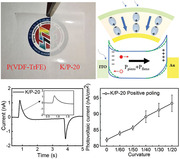

## 1 Introduction

With the rapid growth in demand for ferroelectric random access memory (FeRAM) in wearable electronics, the photocurrent from the anomalous photovoltaic (APV) effect of ferroelectric materials has been used for the nondestructive readout of FeRAM.^[^ ^1^ ^]^ The APV effect widely exists in ferroelectric semiconductor materials, including barium titanate (BTO),^[^ ^2^, ^3^ ^]^ lead zirconate titanate (PZT),^[^ ^4^, ^5^ ^]^ and silver niobate (AgNbO_3_).^[^ ^6^ ^]^ This phenomenon derives from excited electrons spanning the impurity level to the conduction band due to the interaction between phonons and electrons. In this phenomenon, electrons can be excited by light illumination, resulting in a stable photocurrent in an external circuit.^[^ ^7^ ^]^ In the 1970s, the APV effect was discovered in an iron‐doped potassium niobate (KNbO_3_:Fe) bulk single crystal.^[^ ^8^, ^9^ ^]^ It was found that the photovoltage under a light intensity of 1 W cm^–2^ is a few orders of magnitude larger than the bandgap in a KNbO_3_:Fe single crystal. Later, Sasabe et al. investigated the photovoltaic current (*I*_pv_) in a PVDF (poly(vinylidene fluoride) film and found that the *I*_pv_ is much stronger than the dark current in the PVDF film.^[^ ^10^ ^]^ In 2016, Verkhovska et al. investigated the photovoltaic current in PVDF‐TrFE (Poly(vinylidene fluoride‐trifluoroethylene)) film and found a light conversion efficiency of 0.02% for PVDF‐TrFE with carbon nanotube film.^[^ ^11^ ^]^ However, this efficiency was much lower than that of solar cell devices. Therefore, improving the polarization in PVDF‐based materials and eventually enhancing the light conversion efficiency of ferroelectric materials is a critical issue for future applications in the nondestructive read‐out of ferroelectric memory and photocurrent devices.

Since the development of electric polarization generated from curvature strains in liquid crystals by R. B. Meyer in the 1960s,^[^ ^12^ ^]^ flexoelectricity has attracted enormous attention in scientific fields because there is no prerequirement of an asymmetric structure in the material to generate polarity.^[^ ^13^, ^14^ ^]^ In general, flexoelectricity in a material is generated by strain gradients. Thus, unlike the piezoelectric effect, the flexoelectric effect widely exists in all dielectric materials, even those with symmetric structures. In the 2000s, Cross and his coworkers studied flexoelectricity in a variety of piezoelectric materials.^[^ ^15^, ^16^, ^17^, ^18^ ^]^ Recently, with the development of nanomaterials, especially materials with thicknesses in the nanometer range, a great deal of research has been devoted to studying the flexoelectricity in materials with nanostructures.^[^ ^19^, ^20^ ^]^ Since 2011, Catalan and his colleagues thoroughly investigated the flexoelectricity‐based polarization in the PbTiO_3_ ‐based ferroelectric thin film and the PbZrO_3_‐based antiferroelectric thin film.^[^ ^21^, ^22^ ^]^ In 2019, Yang et al. at Alexe's group found that the nonpiezoelectric SrTiO_3_ thin film can easily generate a large strain gradient in the out‐of‐plane direction, causing a large flexo‐polarization, which can significantly affect the photovoltaic effect on the SrTiO_3_ thin films.^[^ ^23^ ^]^ Recently, Shu et al. at Catalan's group studied the photoflexoelectric effect on the perovskite CH_3_NH_3_PbBr_3_ film and found that under a combination of light illumination and mechanical vibration, the film can generate orders of magnitude higher flexoelectricity than in the dark.^[^ ^24^ ^]^

In this investigation, the flexoelectricity generated from a bending curvature is used to improve the photovoltaic current of PVDF‐based nanocomposites. Generally, flexoelectric polarization is expressed by the following equation:

(1) $$\;P_{i}=\mu_{ijkl}\;\frac{\partial \varepsilon_{kl}}{\partial x_{j}}$$

where *P_i_* represents the flexoelectric polarization, *μ_ijkl_* represents the flexoelectric coefficient and $\frac{\partial \varepsilon_{kl}}{\partial x_{j}}$ represents the strain gradient. In 2001, Ma et al. investigated flexoelectric coefficients for various ceramic materials, including lead magnesium niobate (Pb(Mg_1/3_ Nb_2/3_)O_3_), ^[^ ^15^ ^]^ lead zirconium titanate (PZT),^[^ ^25^ ^]^ and BTO.^[^ ^17^ ^]^ In 2014, Jiang's group fabricated Ba_0.7_Sr_0.3_TiO_3_ (BST)‐based thin film (thickness: 130 nm) and investigated the flexoelectric effect of the film.^[^ ^26^ ^]^ Based on that, a group of studies were conducted on the flexoelectricity of BST‐based ceramics.^[^ ^27^, ^28^, ^29^ ^]^ Although ceramic materials can exhibit a relatively large flexoelectricity coefficient *μ_12_* (1.4−10 µC m^−1^), generating a large strain gradient remains a challenge. Due to their flexibility and capability of generating a large strain, piezoelectric polymers have attracted considerable attention in this research area. In 2012, Chu's^[^ ^30^ ^]^ group measured the flexoelectric coefficient *μ_12_* of poly(vinylidene difluoride) (PVDF), indicating that the *μ_12_* is on the order of 10^–9^‐10^–8^ C m^–1^. However, the flexoelectric polarization of PVDF is much lower than that of piezoelectric ceramic materials. In 2018, Chu's group studied the flexoelectric coefficient of a barium strontium titanate/PVDF (BST/PVDF) nanocomposite film and found that the flexoelectric coefficient *μ_12_* of the nanocomposite was 3–4 times higher than that of PVDF film.^[^ ^31^ ^]^ This result demonstrated that piezoelectric ceramic nanoparticles in a PVDF film can improve the flexoelectric coefficient of the nanocomposite material.

In this study, (K_0.5_Na_0.5_)NbO_3_‐LiSbO_3_ (KNN) nanoparticles^[^ ^32^ ^]^ were mixed into a PVDF‐TrFE polymer solution to fabricate KNN/PVDF‐TrFE nanocomposite films.^[^ ^33^ ^]^ The nanocomposite film exhibited a higher polarization, which may benefit the generation of photovoltaic current. We believe that the interface between the KNN nanoparticles and PVDF–TrFE film may play a critical role in the enhancement of flexoelectricity in the nanocomposite film, which can improve the photovoltaic current in the KNN/PVDF‐TrFE nanocomposite. However, it was found that the photovoltaic effect induced by flexoelectricity is higher than the increasing polarization of flexoelectricity, so this effect is called as the flexophotovoltaic effect. This is the first investigation of the flexophotovoltaic effect in a KNN/PVDF‐TrFE nanocomposite. The results show great promise for the applications of nondestructive readout ferroelectric memory and energy harvesting devices.

## 2 Results

### 2.1 Characterization of Composite Films

**Figure 1:**
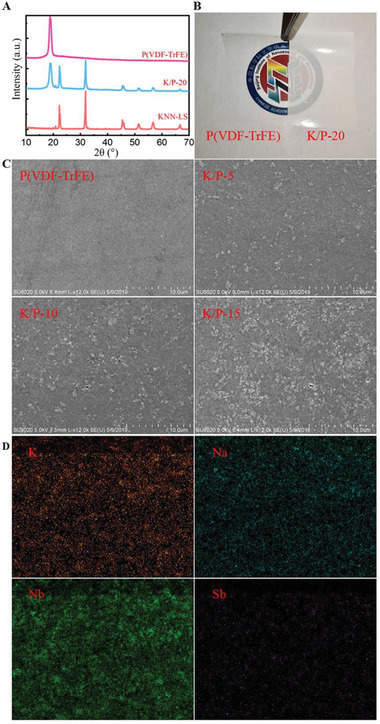
A) X‐ray diffraction (XRD) patterns of the pristine (poly(vinylidene fluoride‐trifluoroethylene)) (PVDF‐TrFE) film, K/P‐20, and KNN particles. B) Digital image of pristine PVDF‐TrFE and K/P‐20. C) scanning electron microscopy (SEM) images of the composite films with various KNN concentrations (pristine, 5%, 10%, 15%). D) SEM image and energy dispersive spectroscopies of K/P‐20.

The X‐ray diffraction (XRD) patterns of PVDF‐TrFE and 0.948 (K_0.5_Na_0.5_)NbO_3_‐0.052 LiSbO_3_ (KNN) nanoparticles and the nanocomposite films were acquired by an X'Pert3 Powder diffractometer (PANalytical, Netherlands) with CuK*α* radiation in the 2*θ* range of 10°–70°. The XRD patterns [**Figure** **1**a] indicate that a characteristic peak at 2*θ* of 20° represents the *β* phase of (110, 200) in the PVDF‐TrFE film.^[^ ^30^, ^31^ ^]^ Additionally, the characteristic peaks at 20.89°, 31.9°, and 45.4° represent the (001, 100), (101, 110), and (002, 200) crystal planes of the KNN nanoparticles, respectively.^[^ ^28^ ^]^ This result indicates that the KNN particles were well distributed inside the nanocomposite film. Furthermore, scanning electron microscopy (SEM) and energy dispersive spectroscopy (EDS) were conducted using a SU8020 microscope (Hitachi Ltd., Japan) to characterize the surface morphology and element distribution of the nanocomposite film. The SEM images (Figure 1c) correspond to the KNN/PVDF‐TrFE composite films with the KNN nanoparticles at various concentrations, including 0%, 5 wt%, 10 wt%, and 15 wt% (hereinafter represented by pristine PVDF‐TrFE, K/P‐5, K/P‐10, and K/P‐15). The images imply that the particle sizes are approximately 200–300 nm and that these nanoparticles are uniformly dispersed in the nanocomposite. The EDS results [Figure 1d] obtained from KNN/PVDF‐TrFE (20 wt%) (K/P‐20) composite film further demonstrate that the KNN particles exist and are well distributed in the composite film. Additionally, the SEM images show that the nanocomposite film exhibits no holes or defects on the surface. This will help reduce the large leakage current of the nanocomposite film.

The Young's moduli and mechanical losses of the KNN/PVDF‐TrFE composite films at various KNN concentrations were measured using a dynamic mechanical analyzer (DMA, TA Instruments Inc., DE, USA). As the data shown in Figure S1A (Supporting Information), the Young's modulus of the nanocomposite changes from 783 MPa to 1.143 GPa at room temperature, with the KNN concentration increasing from 0% to 20%. Although the Young's modulus of the K/P‐20 nanocomposite is increased, it remains relatively small compared to that of KNN ceramic (65 GPa). It demonstrates the flexibility of the nanocomposite films.

**Figure 2:**
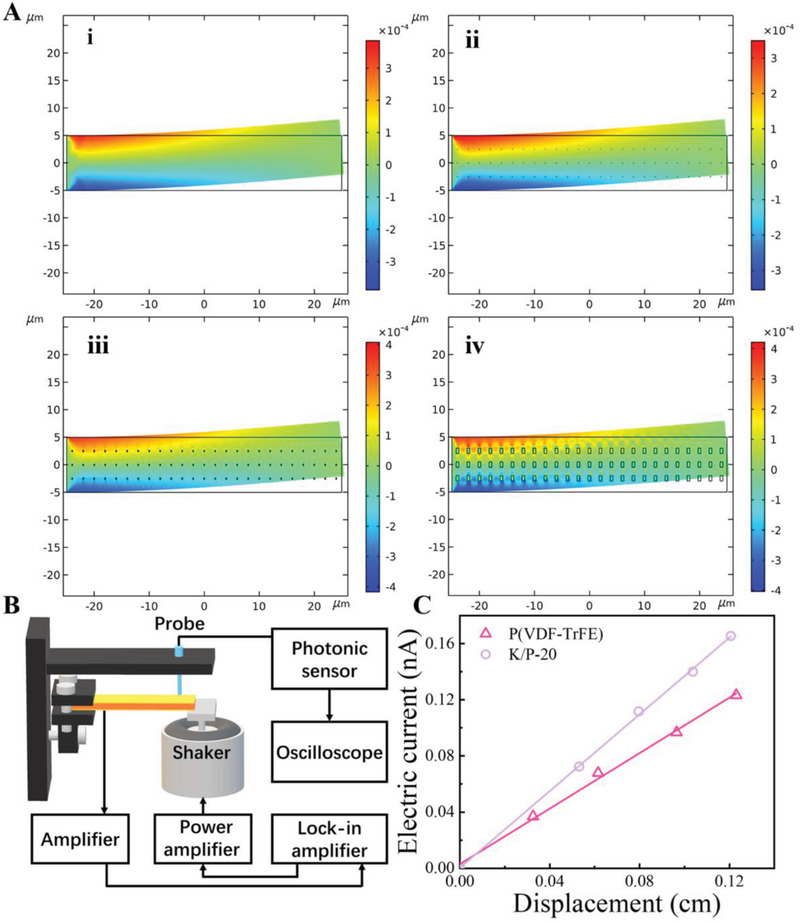
A) Simulated displacement gradient of the nanocomposite using the finite element method. B) Schematic drawing of the flexoelectric coefficient measurement system. C) Electric current induced by the flexoelectric effect as a function of the displacement (w(x)) of the free end of the nanocomposite beam.

To measure the flexoelectric coefficient of the films, a cantilever flexoelectric coefficient measurement system was built in our laboratory as shown in **Figure** **2**B, during the measurement, one end of the nanocomposite film was attached to a sample holder in the system. The other end of the sample was kept free and driven by a shaker. A lock‐in amplifier was used to drive the shaker and generate an up‐and‐down motion and record the output current signal of the film. The photonic sensor was used to measure the displacement of the end of the film. The ratio of flexoelectric current and displacement of the nanocomposite can be defined by the equation below ^[^ ^29^ ^]^

(2) $$i\;=\frac{2\pi f\mu_{12}bL}{x^{2}\left(1-\frac{x}{3L}\right)}\;w\left(x\right)$$

where *i* is the flexoelectric current, *f* is the frequency of the applied force, *b* and *L* are the width and length of the nanocomposite, respectively; *x* is the distance from the fixed end to the measurement point of the nanocomposite film, *w(x)* is the displacement at the free end of the nanocomposite film and *μ*_12_ is the flexoelectric coefficient.

The data shown in Figure 2B indicate that the flexoelectric current is proportional to the tip displacement of the nanocomposite‐based cantilever beam. As the KNN concentration increases from 0% to 20%, the flexoelectric coefficients increase from 3.47 to 4.49 nC m^−1^, which is improved by ≈29%. It is believed that in case of bending, the interface between the PVDF‐TrFE and KNN particles can generate large deformation due to the huge difference in the moduli of those two materials. The effect of the interface between PVDF‐TrFE and KNN nanoparticles plays an important role in improving the flexoelectric coefficient. This means that the polarization of the nanocomposite film can be greatly improved with additional KNN nanoparticles.

Furthermore, the finite element analysis was conducted using COMSOL Multiphysics software to analyze the various strain gradients on nanocomposites. The nanocomposites were analyzed with various sizes of nanoparticles including 0.1 × 0.05, 0.3× 0.15, and 1× 0.5 µm. As the simulation results shown in Figure 2A, the displacement gradient of the pristine PVDF‐TrFE film is ≈7.73 × 10^–4^, which is similar to that of the nanocomposite with 0.1 µm nanoparticles. If the size of nanoparticles is increased to 300 nm, the simulated displacement gradient increases to 8.26 × 10^–4^, which is similar to that of the nanocomposite with 1 µm nanoparticles. It means that additional nanoparticles between 300 nm to 1 µm can increase the displacement gradient by ≈6.9%. However, relatively large particles in the nanocomposite may significantly reduce the breakdown field of the nanocomposite during the poling process and eventually reduce the polarization of the material. Thus, the particle size within the nanocomposite was chosen as 300 nm in this study. However, with regard to the effect of the nanoparticle on the flexoelectricity of the nanocomposite, the increasing percentage of the simulation result is lower than the experimental value, which can result from the nonuniform distribution of KNN nanoparticles in the PVDF‐TrFE substrate.

To measure the piezoelectric coefficient *d*_33_ of the nanocomposite, the sample was characterized by a lab‐designed experimental setup and a schematic drawing of the experimental setup is shown in Figure S2A (Supporting Information). The piezoelectric coefficient *d*_33_, as a function of applied force in the nanocomposite, is shown in Figure S2B (Supporting Information). The data indicate that the piezoelectric coefficient *d*_33_ of the nanocomposite film increases (from 10.20 to 32.56 pC N^−1^) with increasing concentration of KNN nanoparticles in the nanocomposite. This means that the polarization of the nanocomposite film can be greatly improved with additional KNN nanoparticles. Furthermore, to demonstrate the piezoelectric output of the nanocomposite, a K/P‐20‐based cantilever structure was designed in which PET (poly(ethylene terephthalate)) (250 µm) and a double‐layered K/P‐20 nanocomposite film (20 µm) were used as the passive layer and the active layer, respectively. The experimental results (Figure S3, Supporting Information) show that the maximum output power is ≈45 µW at a resonance frequency of 9 Hz. This experimental result demonstrates that the piezoelectric properties of the PVDF‐TrFE film can be increased with the addition of KNN nanoparticles in the material.

### 2.2 Photovoltaic Measurement

**Figure 3:**
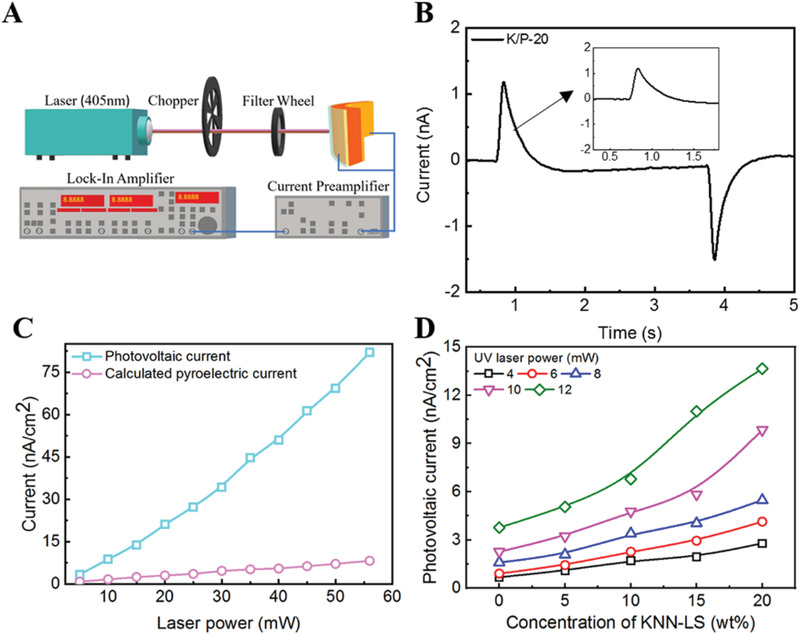
A) Schematic drawing of the experimental setup for photovoltaic current measurement system. B) Time response of photovoltaic current (*I*_pv_) of the K/P‐20 nanocomposite. C) Comparison data between the *I*_pv_ and calculated pyroelectric current of the the K/P‐20 nanocomposite as a function of laser power. D) The *I*_pv_ of the K/P‐20 nanocomposite with various KNN concentrations as a function of laser power.

Under light illumination, if an electric field is applied to a ferroelectric material, the current generated from the APV effect is called the photocurrent (*I*_ph_). Otherwise, if there is no electric field in this process, the generated current is called the photovoltaic current (*I*_pv_). To compare the *I*_pv_ with the pyroelectric current, the photovoltaic current *I*_pv_ of K/P‐20 was measured using the experimental setup shown in **Figure** **3**A. In this measurement, a transparent ITO electrode (indium tin oxide, 3 mm in diameter) was sputter coated as the top electrode of the sample to allow light to pass through it. The time response of the *I*_pv_ of the poled K/P‐20 film was measured, and the data are shown in Figure 3B. This result is in agreement with that of the photovoltaic current derived from H. Sasabe's study^[^ ^10^ ^]^ of PVDF materials. From H. Sasabe's results, it is known that the pyroelectric transient current of PVDF material may include two relaxation processes, which can be characterized by thermal (*τ*_T_) and electric relaxation time (*τ*_E_). From the peak fitting using Das‐Gupa's calculation,^[^ ^10^ ^]^ *τ*_E_ and *τ*_T_ are 0.16 and 0.65 s, respectively. These two relaxation times are consistent with our results. Due to the longer thermal relaxation time, the pyroelectric current mainly consists of the transient current following the peak current. Thus, the negative current after the peak current in Figure 3B may arise from the thermal equilibrium process of the pyroelectric current. Thus, the peak value of the *I*_pv_ is mainly derived from the photovoltaic current.

In addition, the same experimental configuration shown in Figure 3A was used to measure the pyroelectric coefficient of the nanocomposite. During this measurement, the composite film was sputter coated with an Au electrode on top of the sample to avoid the photovoltaic current in this process. The main function of the laser in this experimental process is to generate heat on the nanocomposite film. During this measurement, when a pulse laser [1 s ON, 3 s OFF] was imposed on the nanocomposite, the temperature change of the nanocomposite was measured using a thermocouple glued onto the backside of the samples. After the measurement, the pyroelectric coefficients of the nanocomposites were calculated using the ratio of pyroelectric current density to temperature change ($J_{\mathrm{pyro}}/\left(\frac{\partial T}{\partial t}\right))$. As shown in Figure S3 (Supporting Information), as the concentrations of KNN nanoparticles increase from 0 to 20 wt%, the calculated pyroelectric coefficients of the nanocomposite films increase from 11.27 to 15.32 µC m^–2^ K^–1^. This result is consistent with the piezoelectric coefficients and previous research results for the pyroelectric coefficient of PVDF‐TrFE film (23 µC m^–2^ K^–1^), demonstrating that the polarization of the nanocomposite can increase with the increasing concentration of KNN nanoparticles in the PVDF‐TrFE nanocomposite.

Additionally, the sample temperature was measured for the poled K/P‐20 film as a function of laser power. During this measurement, the sample was sputter coated with ITO and Au as the top and bottom electrodes, respectively, which was same with that of the photovoltaic current measurement. The results shown in Figure S3C (Supporting Information) suggest that the sample temperature can change from 25.8 °C to 28.8 °C if the laser power is increased from 0 to 56.1 mW. Meanwhile, the pyroelectric current was calculated using the polarization equation $p_{\mathrm{pyro}}\left(\frac{\partial T}{\partial t}\right)\;$and the pyroelectric current for the K/P‐20 film is 8.207 nA cm^–2^. However, the data shown in Figure 3C indicate that the *I*_pv_ reached 82.12 nA cm^–2^ if the illuminated laser power was increased to 56.1 mW. This means that the pyroelectric effect accounts for ≈10% of the *I*_pv_ in this application. These measurement results demonstrated that both the pyroelectric effect and APV effect can be generated simultaneously in the light illumination process. However, the photovoltaic current in this process contributes ≈90% of the total current.

Furthermore, the *I*_pv_ values of the poled nanocomposite films with various KNN concentrations were measured, and the results are shown in Figure 3D. The *I*_pv_ data at the same power level indicate that the photovoltaic current density of the nanocomposite gradually increases with increasing KNN concentration. For the sample with the same KNN concentration, the photovoltaic current density of the nanocomposite is proportional to the laser power. At a laser power of 12 mW, the photocurrents of the poled PVDF‐TrFE film and the K/P‐20 nanocomposite are 3.765 and 13.659 nA cm^–2^, respectively. This result demonstrates that 20% KNN nanoparticles can increase the *I*_pv_ of the PVDF‐TrFE film by ≈2.6 times. The data indicate that the nanocomposite with KNN nanoparticles can greatly improve the photovoltaic current in this investigation.

### 2.3 Flexoelectric Effect

**Figure 4:**
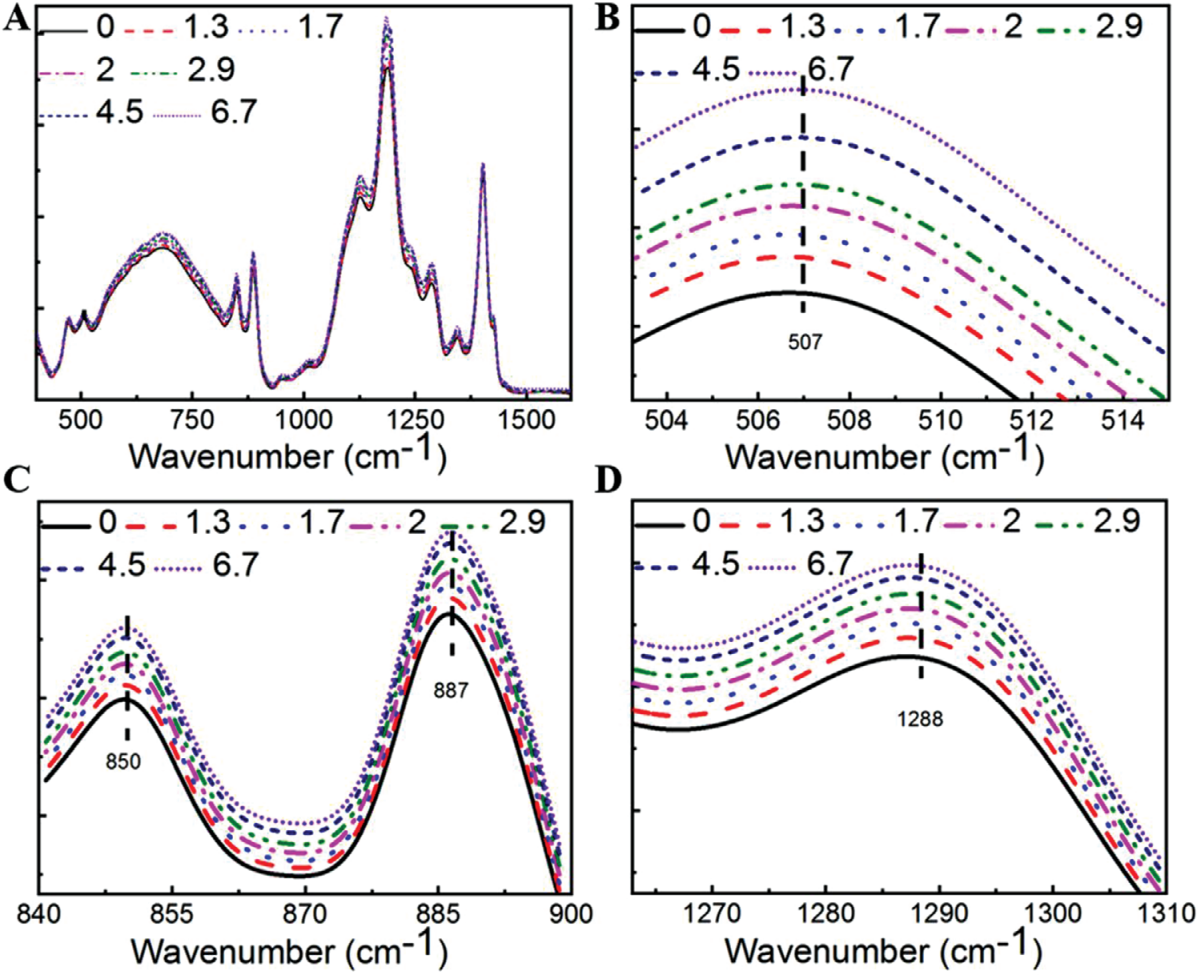
A) Full view of FTIR spectra of the K/P‐20 nanocomposite and the enlarged FTIR intensity peaks at wavenumbers of B) 507 cm^–1^, C) 850 and 887 cm^–1^, and D) 1288 cm^–1^.

In this investigation, a strain gradient was generated to enhance flexoelectricity along the preferred direction to improve the photovoltaic current of the nanocomposite film. To understand how different functional groups in the KNN/PVDF‐TrFE nanocomposite respond to the flexoelectric effect, Fourier transform infrared (FTIR) spectra were measured using a VERTEX 80v FTIR spectrometer (Bruker Inc., MA, USA). As the schematic drawing shows in Figure S4 (Supporting Information), a lab‐designed setup was used in the FTIR spectrometer to change the bending curvature of the nanocomposite film. The FTIR spectra at various bending curvatures are shown in **Figure** **4**. It is known that the absorption peaks at 507 and 850 cm^–1^ correspond to the bending of CF_2_ [*δ*(CF_2_)] and the antisymmetric stretching of CF_2_ [*ν*_as_(CF_2_)] in the T_3_G conformation, respectively, which are assigned to the *β* phase of the PVDF‐TrFE film.^[^ ^31^ ^]^ In addition, the absorption peak at 887 cm^–1^ corresponds to *ν*_as_(CF_2_) and the rocking of the CF_2_ group [*r*(CF_2_)] associated with the rocking of the CH_2_ group in all *trans* conformations. The absorption peak at 1288 cm^–1^ corresponds to *ν*_as_(CF_2_) associated with the antisymmetric stretching [*ν*_as_(CC)] and the bending vibration of the backbone [*δ*(CCC)]. Both absorption peaks at 887 and 1288 cm^–1^ can be affected by the orientation of CF_2_−CH_2_ dipoles in the PVDF‐TrFE film. As shown in Figure 4B−D, the peak intensities at 507, 850, and 887 cm^–1^ bands of the K/P‐20 nanocomposite films were enhanced with increasing bending curvature of the nanocomposite film. The results also demonstrated that bending can effectively change the chain orientation and improve the *β* phase conformation and polarity of the nanocomposite.

**Figure 5:**
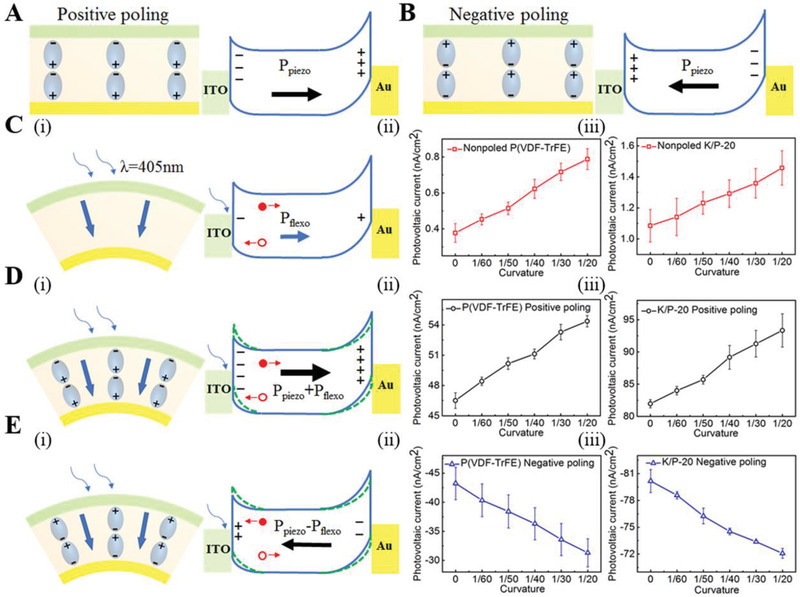
Schematic drawing of the dipole structure and energy band diagram of the nanocomposite with A) positive poling, and B) negative poling, where positive poling represents the nanocomposite polarized with a positive DC electric field on the top ITO electrode; negative poling represents the sample polarized with a negative DC electric field on the top ITO electrode. C) (i) Energy band diagram, (ii) the photovoltaic current (*I*_pv_) the unpoled pristine (poly(vinylidene fluoride‐trifluoroethylene)) (PVDF‐TrFE) film, (iii) the *I*_pv_ of the unpoled K/P‐20 nanocomposite film; D) (i) Energy band diagram, (ii) the *I*_pv_ of the PVDF‐TrFE film with positive poling, and (iii) the *I*_pv_ of the K/P‐20 film with positive poling; E) (i) Energy band diagram, (ii) the *I*_pv_ of the PVDF‐TrFE film with negative poling, and (iii) the *I*_pv_ of the K/P‐20 film with negative poling.

In the testing process of flexoelectricity, a near‐UV laser (405 nm) with a spot size of 3 mm in diameter was used as a light source. The photovoltaic current *I*_pv_ of the pristine PVDF‐TrFE and K/P‐20 samples was measured as a function of bending curvature (0: flat state, 1/*R*: the curvature with a radius of *R* in cm). In this experimental process, a filter wheel was used to control the power of the UV laser, and a lock‐in amplifier was used to record the generated photovoltaic current. As shown in **Figure** **5**Cii andiii, the data indicate that the current densities of the unpoled PVDF‐TrFE film and K/P‐20 composite are 0.37 and 1.08 nA cm^–2^, respectively. Under this condition, excited electrons could be trapped inside the polymer material because electrons have difficulty drifting out from the material without an internal electric field. Thus, a very weak current was generated by laser illumination in the unpoled samples. The data also demonstrate that for an unpoled sample, the current density increases with increasing bending curvature of the samples. At a curvature of 1/20, the *I*_pv_ of the unpoled PVDF‐TrFE film increases from 0.37 to 0.79 nA cm^–2^, which is approximately 2.1 times that of the flat PVDF‐TrFE film. A similar trend occurs for the unpoled K/P‐20 nanocomposite. The *I*_pv_ of the curved K/P‐20 nanocomposite (1/20 curvature) increases from 1.08 to 1.46 nA cm^–2^, which is a 35% increment compared to that of the flat K/P‐20 film. However, compared with the PVDF‐TrFE film with the same curvature (1/20), the *I*_pv_ of the curved nanocomposite film exhibited an ≈85% enhancement. This demonstrates that the nanoparticles inside the composite may induce strain gradients at the interface between the nanoparticles and polymer substrates. This result indicates that flexoelectricity can induce electric polarization, which improves the photovoltaic current of the composite film.

For poled ferroelectric materials, the dipoles in the material are arranged mainly along one direction. Therefore, the ferroelectric material can possess an internal electric field. When a laser is illuminated on the poled ferroelectric film, electrons can be excited from impurity levels to the conduction band, and these electrons can drift rapidly along the direction of the internal electric field to form a photovoltaic current. Thus, the polarization generated from a strain gradient can either enhance or deteriorate the polarization of the ferroelectric film. In this investigation, a strain gradient was generated from the bending of the nanocomposite, if an internal electric field is enhanced by the polarization from flexoelectricity, the photovoltaic current *I*_pv_ will increase. In contrast, the photovoltaic current of the material can be decreased if the polarization is reduced by the flexoelectricity effect. To explore this phenomenon, two samples with opposite polarization directions were poled, and the photovoltaic current of the sample was measured using the same setup. As shown in Figure 5A,B, the sample with dipole orientations from the bottom to the top is labeled poling^+^ (positive poling). The sample with dipole orientation from the top to the bottom is labeled poling^–^(negative poling). The data shown in Figure 5Eii and iii indicate that the bending curvature induces a negative effect on the photocurrent for both the PVDF‐TrFE film and the K/P‐20 nanocomposite with poling**^–^**. At a bending curvature of 1/20, the *I*_pv_ of the K/P‐20 nanocomposite with poling**^–^** can be reduced by ≈10.1% (from 80.17 to 72.07 nA cm^–2^). In contrast, the polarization induced from the bending curvature induced a positive effect on the photovoltaic current for the nanocomposite with poling^+^. As shown in Figure 5Dii, for the PVDF‐TrFE film with poling+, the *I*_pv_ of the curved PVDF‐TrFE film (1/20 curvature) can increase by ≈17% (from 46.51 to 54.4 nA cm^–2^) compared to that of the flat PVDF‐TrFE film. As a comparison, the *I*_pv_ of the curved K/P‐20 film (1/20 curvature) increases by ≈13.9% (from 81.95 to 93.34 nA cm^–2^) compared to that of the flat K/P‐20 film, which is slightly lower than the increasing percentage of *I*_pv_ in the PVDF‐TrFE film during bending. This could mainly be attributed to the nonuniform distribution of KNN nanoparticles. However, at the same curvature (1/20 curvature), the *I*_pv_ of the curved K/P‐20 nanocomposite is 72% higher (from 54.4 to 93.34 nA cm^–2^) than that of the curved PVDF‐TrFE film. It is believed that the KNN nanoparticles are resistant to twisting and change their shape during the bending process of the nanocomposite. However, these nanoparticles can help increase the curvature of the PVDF‐TrFE film around their interfaces. This demonstrates that the photocurrent derived from the APV effect in PVDF‐based nanocomposites can be greatly improved by flexoelectric polarization.

This effect can be explained using the energy band diagrams shown in Figure 5. Due to the difference in the work function between Au and ITO, the energy barrier at the PVDF‐TrFE/Au interface will be higher than that of the PVDF‐TrFE/ITO interface. Therefore, the energy band structure for the nanocomposite with positive poling and negative poling are schematically drawn as those shown in Figure 5A,B, respectively. Since the light reaches the interface with ITO first, so that the photovoltaic current is mainly generated at the ITO/PVDF‐TrFE interface, while the interface of PVDF‐TrFE/Au generates little photovoltaic current. Therefore, our discussion mainly focuses on the ITO/PVDF‐TrFE interface. In general, the flexoelectric polarization (*P*_flexo_) may modulate the barrier heights of the two end contacts and influence the *I*_pv_ of the nanocomposite (Figure 5C). As shown in Figure 5Di, for the nanocomposite film with positive poling, the flexoelectric polarization from bending may increase the barrier height of the PVDF‐TrFE/ITO interface owing to the adding up of the piezoelectric effect and flexoelectric effect. In this case, the flexoelectric polarization is in the same direction with the piezoelectric polarization, thus it can increase the internal electric field in the nanocomposite and induce a large photovoltaic current in the external circuit. At the interface, the photon generated electrons move toward the Au electrode, while the holes move toward ITO. In contrast, for the nanocomposite with negative poling, the *P*_flexo_ is still pointing to the PVDF/Au interface and it will increase the barrier height of the PVDF/ITO interface as shown in Figure 5E. However, under this condition, the *P*_flexo_ is in the opposite direction with the piezoelectric polarization in the nanocomposite, which can reduce the internal electric field in the nanocomposite. Thus, the flexoelectric polarization will decrease the *I*_pv_ of the nanocomposite for the nanocomposite film with negative poling. Due to the change in the polarization direction, at the interface, the photon generated electrons move toward the ITO electrode, while the holes move toward Au.

**Figure 6:**
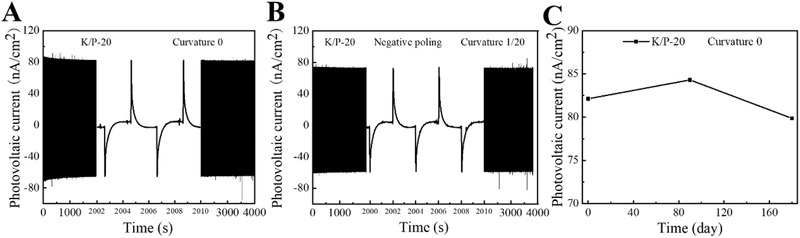
Stability testing of K/P‐20 film. A) 1000 cycles under 405 nm laser irradiation (curvature 0). B) 1000 cycles under 405 nm laser irradiation (curvature 1/20). C) Reusability testing.

Furthermore, the stability of the nanocomposite with a curvature of 1/20 was evaluated by measuring the *I*_pv_ of the nanocomposite at a laser power of 56 mW for 1000 times (the measurement period: 66 min). The data shown in **Figure** **6**A indicate that the *I*_pv_ of the sample was approximately 73 nA cm^−2^ during the measurement process. The measurement data demonstrated the stability of the K/P‐20 nanocomposite for the photovoltaic effect. In addition, it was found that the failure of the samples were mainly due to the broken ITO electrode or the depolarization caused by the high temperature (>65 °C) of the high power laser. Otherwise, the output current of the nanocomposite is very stable. Furthermore, from the measurement data shown in Figure 6C, it was found that the *I*_pv_ of the K/P‐20 nanocomposite only attenuated for 2.5% (from 82 to 79 nA cm^−2^) after 3‐month period, which proved the great resuability of the K/P‐20 nanocomposite sample for future applications of photovoltaic effect. This investigation shows that KNN/PVDF‐TrFE based nanocomposites hold great promise for nondestructive readout of FeRAM applications.

## 3 Discussion and Conclusion

In this investigation, due to a relatively large band gap (≈6 eV) in PVDF‐based ferroelectric film, the Schottky effect is unexpected in the polymer nanocomposite. Therefore, it is believed that the APV effect plays a critical role in this study. The relation between light intensity and photovoltaic current *J*_pv_ can be expressed using the following equation:^[^ ^11^ ^]^

(3) $$\;J_{pv}=\;\alpha GI$$

where *G* is the Glass coefficient,^[^ ^34^ ^]^ *I* is the intensity of light, and *α* is the absorption coefficient of the ferroelectric film, which can be defined by the Beer‐Lambert relation of *α* = 2.303 A t^−1 [^ ^33^ ^]^ (in this equation, *A* is the absorbance, which was measured using a UV‐3600UV‐VIS‐NIR spectrophotometer (Shimadzu Corp., Kyoto, Japan), and the data are shown in Figure S5 (Supporting Information), where *t* is the thickness of the nanocomposite film). The electric field generated from the APV effect can be expressed as:

(4) $$\;E_{pv}=J_{pv}\;/\left(\sigma_{d}+\sigma_{ph}\right)$$

where *σ*_ph_ represents the photoconductivity and can be calculated by *σ*_ph_ = t*I/V*S (*S* is the illumination area of light on the film, *V* is the applied voltage on the film). *σ*_d_ is the dark conductivity of the ferroelectric film. In this investigation, the dark current *σ*_d_ was measured using a high‐voltage leakage current test system PK‐SPIV17T (PolyK Technologies LLC, PA, USA), and the data are shown in Figure S5 (Supporting Information). From the data, it can be seen that the photocurrent *I*_ph_ is much larger than the dark current of the nanocomposite. Thus, the electric field derived from the APV effect can be directly calculated by *J*_pv_/*σ*_ph_. In this application, the calculated *E*_pv_ (with a laser power of 1 W) for the poled K/P‐20 nanocomposite film is 3.5 × 10^7 ^V m^–1^, and the open circuit voltage is 630 V, which is much higher than the band gap of the PVDF material (6 eV). Finally, the conversion efficiency of light to electrical energy in ferroelectric material can be expressed as:

(5) $$\eta \;=\;GE_{\mathrm{pv}}$$

From the measurement results of K/P‐20, the absorption coefficient, the thickness of the sample, the Glass constant, and the light energy density are 278 cm^–1^, 18 µm, 3.7 × 10^–12^ m V^‐1^, and 0.79 W cm^–2^. Therefore, the calculated efficiency of the flat K/P‐20 film is 0.013%. At a curvature of 1/20, the energy conversion efficiency of the K/P‐20 nanocomposite film can reach 0.017%, which is comparable to the previous results. This result is also confirmed by the measured flexoelectric coefficients. Although this efficiency is lower than that of solar cell devices, it opens a new approach to improving the efficiency of the APV effect in piezoelectric materials.

In summary, we demonstrated that the internal electric field generated from the flexoelectricity plays a critical role in the APV effect of KNN/PVDF‐TrFE nanocomposite rather than the modulation of energy barrier in the material. From the experimental results, the total photovoltaic current of the curved nanocomposite can increase 13.6% compared to that of the nanocomposite under flat conditions. At a curvature of 1/20, the photovoltaic current density *J_pv_* of the K/P‐20 nanocomposite film reached 93.34 nA cm^‐2^, which is 85% higher than that of the PVDF‐TrFE film. Thus, this new effect is called as the flexophotovoltaic effect. The measured energy conversion efficiency was 0.017%, which is comparable to the previous results. ^[^ ^11^ ^]^ This result is also confirmed by the measured flexoelectric coefficients. Although this efficiency is lower than that of solar cell devices, it opens a new approach to improving the efficiency of APV effect in piezoelectric materials.

## 4 Experimental Section

##### Fabrication of the Nanocomposite Films

In this experiment, lead‐free KNN nanoparticles were prepared using a solid‐state reaction method and used as ceramic fillers in the KNN/PVDF‐TrFE nanocomposite. Before the experimental process, potassium carbonate (K_2_CO_3_, 99%), lithium carbonate (Li_2_CO_3_, 99.8%), and niobium oxide (Nb_2_O_5_, 99.8%) were purchased from the Aladdin Industrial Corp. Sodium carbonate (Na_2_CO_3_, 99.8%) was purchased from Heowns Biochem Technologies, LLC (Tianjin, China), and antimonic oxide (Sb_2_O_5_, 99.5%) was purchased from John Long Technologies (Beijing, China). During the nanoparticle fabrication process, certain amounts of K_2_CO_3_, Na_2_CO_3_, Nb_2_O_3_, Li_2_CO_3_, and Sb_2_O_3_ (1.66:1.28:6.32:0.09:0.37) were weighed following the stoichiometry method and milled with anhydrous ethanol solution in a mill grinder machine for 24 h to obtain uniform raw material powders. Afterwards, the raw material powders were poured into a crucible and calcined at 850 °C for 6 h and then cooled at a rate of 5 °C min^–1^ to room temperature. Next, the calcined KNN particles were milled with anhydrous ethanol in a mill grinder machine again for 48 h to obtain KNN nanoparticles with a size of ≈300 nm. Then, the KNN/PVDF‐TrFE nanocomposite film was prepared by a solution casting method in which 0.2 g of PVDF‐TrFE powder was dissolved in DMF and fully stirred for 4 h to obtain a uniform polymer solution. Next, a certain amount of KNN powder was poured into the PVDF‐TrFE solution and stirred for 4 h to obtain the KNN/PVDF‐TrFE mixture solution. Next, the mixture was poured onto a glass slide and heated in an oven at 65 °C for 10 h to obtain a uniform KNN/PVDF‐TrFE film. Finally, the composite film was annealed at 130 °C for 4 h to improve the crystallinity of the nanocomposite film.

##### Sample Preparation

For the piezoelectric coefficient *d*_33_ test, double‐sided Au electrodes were sputter coated onto a film by a sputter coater machine. After that, the composite was poled to 40 MV m^–1^ for 2 h at 55 °C. To prepare the sample for the photovoltaic current measurement, the poled sample was sputter coated with the ITO and Au electrodes on the top electrode and bottom of the sample, respectively. In addition, positive polarization and negative polarization samples were obtained using either a positive or a negative DC electric field applied on the top of the samples, respectively.

##### Piezoelectric Coefficient Measurement Setup *d*_33_

The measurement setup of piezoelectric coefficient consists of a VT‐20 shaker (YMC Piezotronics Inc., Jiangsu, China), an XYZ stage, an SRS 830 lock‐in amplifier (Stanford Research Systems Inc., CA, USA), and a load cell (Measurement Specialties Inc., VA, USA). In this system, the shaker was used to apply a mechanical force to the sample, and the load cell and the lock‐in amplifier were used to measure the applied force and the generated charges from the sample during the process, respectively.

## Conflict of Interest

The authors declare no conflict of interest.

## Supporting information

Supporting Information
