# Supplementary material for: Flexophotovoltaic Effect in Potassium Sodium Niobate/Poly(Vinylidene Fluoride‐Trifluoroethylene) Nanocomposite
Source: Adv Sci (Weinh). 2021 Feb 8;8(8):2004554. doi: 10.1002/advs.202004554 (PMC8061384; doi:10.1002/advs.202004554)

© 2021 Wiley-VCH GmbH

ADVANCED SCIENCE
Open Access

Supporting Information

for *Adv. Sci.,* DOI: 10.1002/advs.202004554

Flexophotovoltaic effect in potassium sodium niobate/poly(vinylidene fluoride-trifluoroethylene) nanocomposite

Chenchen Wang, Yang Zhang, Bowen Zhang, Bo Wang, Jinxi Zhang, Long-Qing Chen, Q. M. Zhang, Zhong Lin Wang, Kailiang Ren**

Supporting Information

Flexophotovoltaic effect in potassium sodium niobate/poly(vinylidene fluoride-trifluoroethylene) nanocomposite

Chenchen Wang, Yang Zhang, Bowen Zhang, Bo Wang, Jinxi Zhang, Long-Qing Chen, Q. M. Zhang, Zhong Lin Wang, Kailiang Ren**

C. Wang, B. Zhang, J. Zhang, Prof. Z. L. Wang, Prof. K. Ren

Beijing Institute of Nanoenergy and Nanosystems, Chinese Academy of Sciences, Beijing 100083, P. R. China.

E-mail: renkailiang@binn.cas.cn; zlwang@binn.cas.cn.

Y. Zhang

Institute of Semiconductors, Chinese Academy of Sciences, Beijing 100083, P.R. China.

B. Wang, Prof. L. Q. Chen

Department of Materials Science and Engineering, The Pennsylvania State University, University Park, PA 16802, USA.

Prof. Q. M. Zhang

Department of Electrical Engineering and Materials Research Institute, Pennsylvania State University, University Park, PA 16802, USA.

Prof. Z. L. Wang

School of Material Science and Engineering, Georgia Institute of Technology, Atlanta, GA 30332, USA.

Prof. K. Ren
School of Physical Science and Technology, Guangxi University, Nanning, Guangxi 530004, P.R. China.

Figure S1. (A). Young's moduli and (B) mechanical losses of the KNN/PVDF-TrFE composite films with different KNN concentrations.

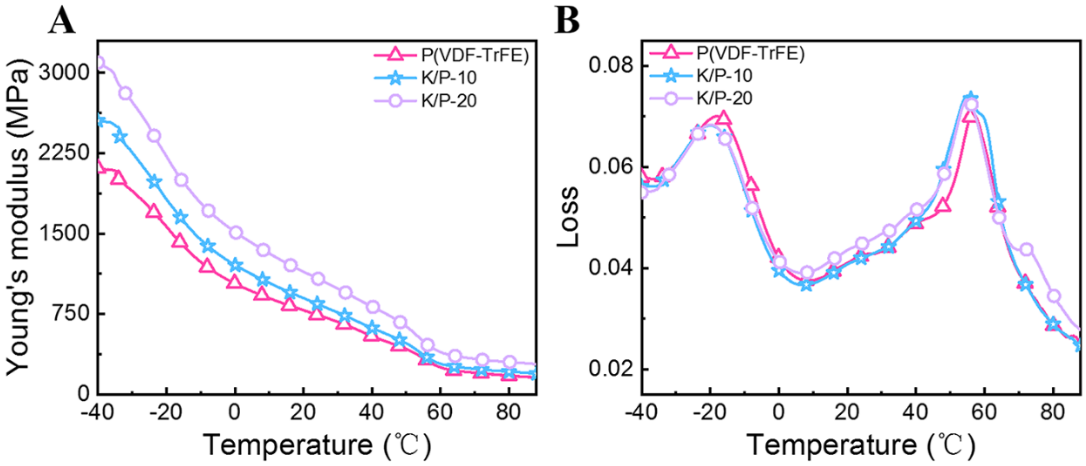

Figure S2.

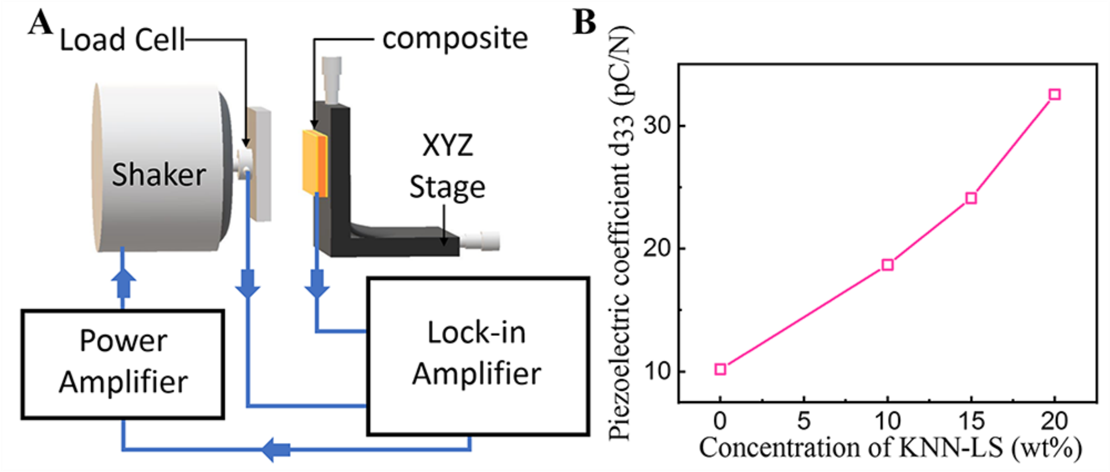
A
Load Cell
composite
Shaker
XYZ
Stage
Power
Amplifier
Lock-in
Amplifier
B
Piezoelectric coefficient d33 (pC/N)
Concentration of KNN-LS (wt%)
0
5
10
15
20
10
20
30

Figure S3. (A) Schematic drawing of a cantilever testing system for the KNN/PVDF-TrFE nanocomposite. (B) Output performance of the K/P-20 nanocomposite film.

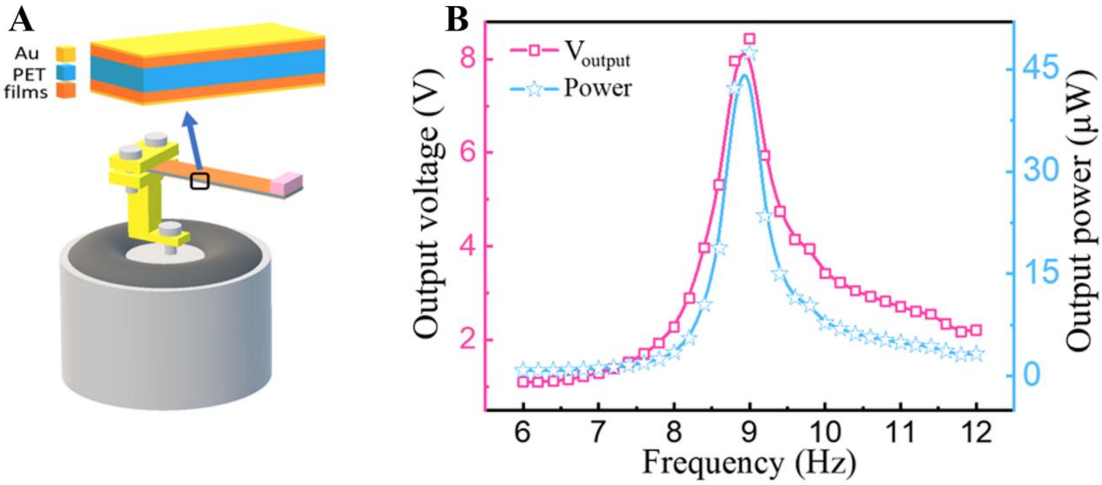

Figure S4. Pyroelectric current of (A) the K/P-20 nanocomposite, (B) the pristine PVDF-TrFE film. (C) Temperature change of the K/P-20 nanocomposite film as a function of laser power.

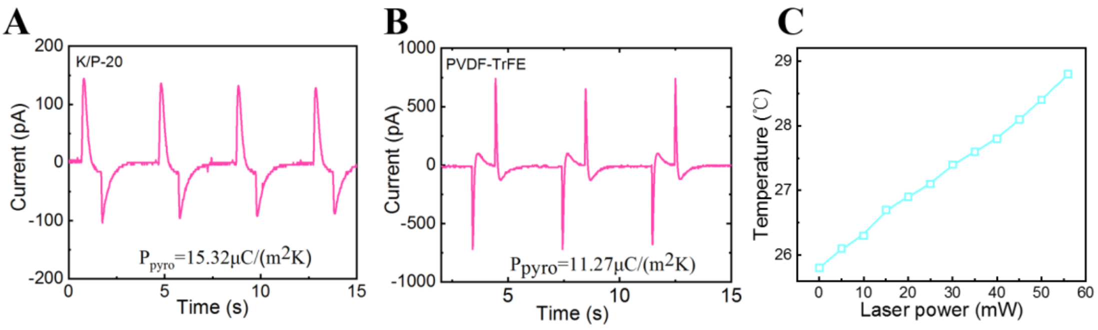

Figure S5. Schematic diagram of the manual slide used in the FTIR test to change the curvature of the nanocomposite film.

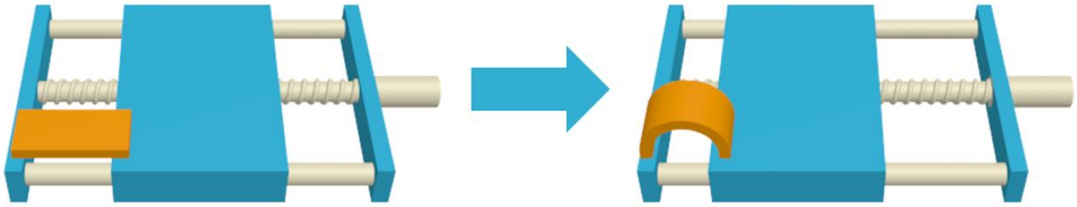

Figure S6. (A) Comparison data between the photocurrent (I_{ph}) and dark current of K/P-20. (B) Optical absorption spectrum of the K/P-20 nanocomposite film.

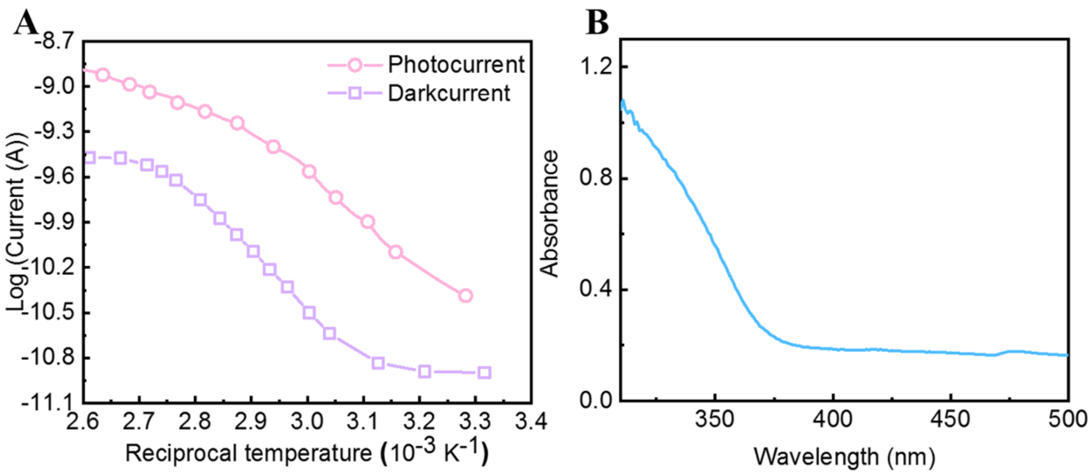

Supplement: Supplementary file 1 — Supporting Information [file ADVS-8-2004554-s001.pdf]
